# Health-related quality of life in older depressed psychogeriatric patients: one year follow-up

**DOI:** 10.1186/s12877-016-0310-6

**Published:** 2016-07-07

**Authors:** Anne-Sofie Helvik, Kirsten Corazzini, Geir Selbæk, Guro Hanevold Bjørkløf, Jerson Laks, Jūratė Šaltytė Benth, Truls Østbye, Knut Engedal

**Affiliations:** Department of Public Health and General Practice, Faculty of Medicine, Norwegian University of Science and Technology (NTNU), Trondheim, Norway; St. Olav’s University Hospital, Trondheim, Norway; Norwegian National Advisory Unit on Aging and Health, Vestfold Health Trust, Tønsberg, Norway; Duke University School of Nursing, Durham, NC USA; Centre for Old Age Psychiatric Research, Innlandet Hospital Trust, Ottestad, Norway; Faculty of Medicine, Institute of Health and Society, University of Oslo, Oslo, Norway; Institute of Psychiatry, Federal University of Rio de Janeiro, Rio de Janeiro, Brazil; Post Graduation Program in Translational Medicine, Universidade do Grande Rio (Unigranrio), Rio de Janeiro, Brazil; Institute of Clinical Medicine, Ahus Campus, University of Oslo, Oslo, Norway; Research Centre, HØKH, Akershus University Hospital, Lørenskog, Norway; Duke Global Health Institute, Durham, NC USA

## Abstract

**Background:**

Knowledge about long-term change in health related quality of life (HQoL) among older adults after hospitalization for treatment of depression has clinical relevance. The aim was firstly to describe the change of HQoL one year after admission for treatment of depression, secondly to explore if improved HQoL was associated with remission of depression at follow-up and lastly to study how HQoL in patients with remission from depression were compared to a reference group of older persons without depression.

**Method:**

This study had the one year follow-up information of 108 older patients (≥60 years), all hospitalized for depression at baseline, and a reference sample of 106 community-living older adults (≥60 years) without depression. HQoL was measured using the EuroQol Group’s EQ-5D Index and a visual analog scale (EQ-VAS). Depression and remission were diagnosed according to ICD-10. Socio-demographic variables (age, gender, and education), depressive symptom score (Montgomery-Aasberg Depression Rating Scale), cognitive functioning (Mini Mental State Examination scale), instrumental activities of daily living (the Lawton and Brody’s Instrumental Activities of Daily Living Scale), and poor general physical health (General Medical Health Rating) were included as covariates.

**Results:**

HQoL had improved at follow-up for the total group of depressed patients, as indicated by better scores on the EQ-5D Index and EQ-VAS. In the multivariate linear regression model, improved EQ-5D Index and EQ-VAS was significantly better in those with remission of depression and those with better baseline physical health. In adjusted analyses, the HQoL in patients with remission from depression at follow-up did not differ from the HQoL in a reference group without depression.

**Conclusion:**

Older hospital patients with depression who experienced remission one year after admission gained HQoL and their HQoL was comparable with the HQoL in a reference group of older adults without depression when adjusting for differences in socio-demographics and health conditions.

## Background

Depression is one of the most common mental disorders among older adults [1], and the most common mood disorder in late life, which has led to increasing global concern as the number of older adults rises [2, 3]. About 4 to 10 % of older adults in community settings suffer from major depressive disorders [3, 4]. Risk factors for depression among older adults include female gender, physical morbidity, and an impaired level of physical and cognitive functioning [5]. Depression has been associated with an increased risk of mortality [5, 6], poorer outcome of treatment of physical disorders [7, 8],and health related quality of life in older adults [9].

Health related quality of life (HQoL) relates to the perceived effects of mental and physical health on each individual’s ability to live a fulfilling life [10, 11]; thus, it is an important outcome measure in old age [12–14] and a multidimensional concept [10, 15]. It exists no consensus on the definition of HQoL or the questionnaires to use [16]. In the present study, HQoL was understood as a simple index formed by an algorithm where the importance of five health problems (mental and physical) were taken into account and a visual analog scale where experiences of overall health were rated [17].

Knowledge about change in HQoL after treatment of depression among older adults is an important outcome-measure of relevance for those with depression as well as their family, health care planners, and health care providers. A recent review, identified five longitudinal studies of depressed psychogeriatric in- and outpatients [9] with no follow up after discharge [18–21] or up to 3 months after discharge [22]. Higher severity of depressive symptoms at baseline was related to poorer HQoL at discharge [18, 20] and 3 month follow-up [22]. One study have reported that the HQoL was significantly better in older adults with remission compared to older adults without remission [19]. However, this was an outpatient study without a follow-up period after discharge. Studies using a follow-up after discharge are needed to better understand how remission of depression may influence change of HQoL over time.

It is known that older patients with depression may both have poorer baseline and follow-up physical health and HQoL than older adults without depression [21, 23]. However, as far as we know, if HQoL in patients with remission after treatment using a long-term follow-up perspective is comparable to those without depression when adjusting for physical and mental health differences has yet to be studied [9]. This is highly warranted and clinically relevant information.

The aim of the present study was first to describe HQoL by the EQ-5D Index and EQ-VAS in patients with depression when first admitted to a psychogeriatric unit and then 1 year after admission. Secondly, we wanted to explore if and how improved HQoL 1 year after admission was explained by the remission of depression at follow-up, adjusting for socio-demographic variables and baseline health conditions. Thirdly, we aimed to compare HQoL in the group of patients with remission from depression 1 year after admission with a group of older community living adults without depression, adjusting for differences in socio-demographic and health conditions.

## Method

This study applied data from a 1 year follow-up study to explore the associations between changes of HQoL in hospitalized psychogeriatric patients with depression and remission at follow-up. Furthermore, HQoL data from a cross-sectional study among older community-living adults without depression (reference group) was compared with HQoL data from older patients with remission from depression at follow-up.

### Recruitment, treatment, and follow-up of participants with depression

Patients (aged ≥ 60 years) admitted to one of seven geriatric psychiatry hospital units in Norway between December 1, 2009 and December 30, 2011 with an ICD-10 [24] diagnosis of depression or depressive disorder were evaluated for inclusion in the study sample of depressed older adults. For all potential participants, evaluation of inclusion and exclusion criteria and diagnostic assessments were performed by psychiatrists and psychologists experienced in geriatric psychiatry. Previous and current episodes of depression, together with other mental health problems and family history of mental health problems, were also recorded. Exclusion criteria were severe cognitive impairment defined by a score ≤ 11on the Mini-Mental State Examination-Norwegian Revised Version (MMSE-NR) [25–27], severe aphasia, having a life-threatening medical condition, or inability to complete the questionnaires.

All in-patients admitted to the psychogeriatric hospital units received “treatment as usual” following national guidelines for treatment of diagnosed depression. An interdisciplinary treatment approach, including antidepressant medications, electro-convulsive therapy (ECT), and milieu therapy, was applied.

Included patients were contacted and invited to a follow-up evaluation 12 months after admission at a psychogeriatric out-patient clinic or in the patient’s home by trained project nurses, psychologists, and psychiatrists. The collection of follow-up data was completed February 24, 2014.

In all, 160 patients were included in the study sample. Sixteen patients were excluded from the baseline due to incomplete data, leaving 144 to participate in this study. Three patients died before follow-up, five patients did not consent to participate in the follow-up study, 14 did not fulfill the evaluation of depression one year after admission, and 14 did not answer any of the HQoL measures (EQ-5D Index or EQ-VAS). Thus, 108 participants were included in the follow-up study.

### Recruitment of the reference group

Adults aged ≥ 60 years living in a community setting were recruited for participation in the study through advertisements in the local newspaper, home nursing agencies, senior centers, and volunteer organizations. Potential participants were screened for depressive symptoms and cognitive impairment by trained students of nursing, psychology, and medicine. Diagnostic evaluations were done by an experienced psychologist and physician in geriatric psychiatry. History of mental health problems was recorded. Exclusion criteria included: current depressive episode or depressive disorder according to ICD-10 [24]; current symptoms of other psychiatric disorders; cognitive impairment defined by a score of <27 on the MMSE-NR; severe aphasia; a life-threatening medical condition; inability to complete the study questionnaires; and inability to understand the purpose of the study or to provide informed consent. In total, 215 older adults volunteered to participate in the study, but 47 were excluded because of a MMSE-NR score below 27 points and 56 persons were excluded because of a current ICD-10 depressive episode. Six persons who qualified for study inclusion did not complete any of the required outcome measures; thus, 106 older persons constituted the sample without depression.

### Ethics

This project was approved by the Regional Committee for Ethics in Medical Research in South Eastern Norway. Project nurses informed the inpatients about the research project and invited them to participate. Students in medicine, nursing, and psychology informed potential community-living older adult participants about the study and invited them to participate. Both written and oral information was given before informed consent was signed.

### Measures for patients at baseline and follow-up and the reference group

The health-related-quality of life (HQoL) was assessed using EuroQol Group self-report instruments; ie, including the EuroQol Group EQ-5D descriptive system and the EQ Visual Analog Scale (EQ-VAS). Two separate outcome variables were constructed based the EQ-5D and EQ-VAS.

TheEQ-5D questionnaire asks respondents to grade their mobility, self-care, usual activities, pain/discomfort, and mental health by use of one item for each condition with three possible grades (no problems, some problems, and major problems, ie EQ-5D-3L version of EQ-5D). This allows 243 theoretically possible combinations or situations [17]. A scoring algorithm based on values empirically derived from a large scale U.K. general population sample [17, 28] was used to transform these states into a single utility value (EQ-5D Index) using the “time-trade-off tariff”. The scores on this index range from 0 (death) to 1.0 (best possible situation). Test-retest reliability for the dimensions in the original version of EQ-5D were reported to range from 0.69 to 0.94 [29]. The EQ-5D Index score was used as the first HQoL outcome measure.

The second HQoL outcome measure was the score on the EQ-VAS. This is a standardized 20 cm vertical visual analog scale, with the worst imaginable state as the bottom endpoint (=0) and the best imaginable state as the top outcome (=100). The participants scored their current experienced HQoL by placing a mark at the location that best represented their state. The EQ-5D and EQ VAS were translated, used, and found valid and reliable in Norwegian studies [30], used in studies among adults with depression [31–35] and in older adults with health difficulties other than depression [30, 36, 37].

To rate the severity of depression the Montgomery-Aasberg Depression Rating Scale (MADRS) was used. This is a ten-item rating scale. Each item has a seven-point rating scale from 0 to 6, giving a minimum total score of zero and a maximum score of 60 [38]. A higher score indicates more severe depression. MADRS is validated for older adults in Norway [39], and applied in several Norwegian studies including older adults [40–42].

The Mini-Mental-State Exam-Norwegian Revised Version (MMSE-NR) is a 20-item interviewer-administered assessment scale with scores ranging from0 to 30 points. A score of 27 or more usually indicates healthy global cognitive functioning [25, 27]. The MMSE-NR is a translated, adapted, and validated version of MMSE for older adults in Norway [26, 43].

To assess physical comorbidity The General Medical Health Rating (GMHR), a global rating scale originally used to evaluate medical health in patients with dementia, was applied. One item with four response categories (Excellent-good–fair–poor) [44] comprises the rating scale. The GMHR is highly reliable (weighted kappa = .91), translated, and used in Norwegian studies [45].

The Instrumental Activities of Daily Living (I-ADL) scale by Lawton and Brody [46] was used to evaluate participants’ ability to perform eight instrumental activities of daily living. In this scale, three items were scored from 1 to 3, three items were scored 1–4, and two items were scored 1–5, giving a score range from 8 to 31 points; higher scores indicate a lower level of I-ADL function. The scale is translated and used in several Norwegian studies among older adults [47, 48].

Socio-demographic characteristics (gender, age, and level of education) was assessed by self-report questions that have been used in several studies of older adults in Norway [49, 50].

### Data analysis

The statistical analysis was performed with SPSS version 22.0 (IBMSPSS, Chicago, Ill, USA) and SASv9.3. The comparison between those who were participating at follow-up and those who were not was performed by *χ*^2^- and the Mann-WhitneyU-test. According to graphical assessment of normality, the HQoL outcomes at baseline and follow-up for the total sample were symmetrically distributed, and thus described as mean, standard deviation (SD), and range and compared using a paired-sample test.

Change of HQoL (ie change in the EQ-5D Index and EQ-VAS from baseline to follow-up) was symmetrically distributed in patients with and without remission. Intra-class correlation coefficient (ICC) was used to assess a degree of clustering due to center-level. As no cluster effect was shown, change in HQoL was assessed by estimating linear regression models. Remission versus no remission was the independent variable of primary interest. Socio-demographic information (gender, age, and education) and health-related condition (physical health, MMSE-NR, I-ADL, and MADRS) at baseline were further included as potential confounding variables for change in HQoL.

Also, HQoL assessed by the EQ-5D Index and EQ-VAS were symmetrically distributed in patients with remission at follow-up and in the reference group without depression. No cluster effect on center-level was present in the reference sample, according to ICC. Hence, linear regression models were estimated with the same confounders as in the previous model.

Results with *P*-values ≤ 0.05 were regarded as statistically significant.

## Results

The baseline characteristics of the 108 depressed hospitalized older adults with follow-up information are presented in Table 1. There were no significant differences between those not participating and those participating at follow-up with respect to gender, level of education, history of vascular disease, poor physical health, previous history of psychiatric disease, baseline MMSE, and IADL and MADRS scores. Those not participating were older (mean79.1 SD 6.5 years; *p* < 0.01). The mean duration (SD) from hospitalization to study inclusion was 5.2 (4.7) days, mean duration of hospitalization was 68.2 (48.9) days, and mean duration of follow-up from inclusion was 418.1 (48.2) days. At follow-up, 37 (of 108, 34.2 %) patients had depression, and thus 71 had remitted from depression.

**Table 1:** Characteristics of depressed hospitalized patients at baseline

		Total	
Number of participants		108	(100)
*Socio-demographic variables*			
Females		77	(71.3)
Age (years)	Mean (SD)	75.0	(6.6)
Education ≥ 10 years	N (%)	51	(47.2)
*Health condition*			
History of cardiovascular disease	N (%)	70	(64.8)
GMHR: poor health	N (%)	54	(50.0)
MMSE-NR score	Mean (SD)	26.3	(3.6)
I-ADL score	Mean (SD)	15.2	(6.2)
History of psychiatric problems	N (%)	83	(76.9)
MADRS score	Mean (SD)	26.1	(8.8)

*GMHR*, General medical health rating scale, *MMSE-NR* Mini-Mental State Examination-Norwegian revised version. *I-ADL* Instrumental activities of daily living, *MADRS* Montgomery-Aasberg depression rating scale

### Health related quality of life (HQoL) in the patients

Characteristics of HQoL at baseline and at follow-up are shown in Table 2. The HQoL for the total group was improved at follow-up compared to baseline (*p* < 0.05). In the multivariate linear regression models, being depressed at follow-up was associated with poorer EQ-5D Index and EQ-VAS than in patients with remission (Table 3). Improved EQ-5D Index and EQ-VAS was additionally associated with good baseline physical health. Moreover, improved EQ-VAS was associated with a higher baseline MADRS-score. The explained adjusted variance for EQ-5D Index and EQ-VAS was 27.0 and 27.1 %, respectively.

**Table 2:** Health-related quality of life (HRQoL) (EQ-5D presence of problems, EQ-5D Index, and EQ-VAS score) at baseline and at follow-up stratified by group(depressed- non-depressed) and change in HQoL (EQ-5D Index, and EQ-VAS score) by group

Baseline				Follow-up						Change					
				Total		Depressed		Non-depressed		Total		Depressed		Non-depressed	
Number of participants							37		71						
*EQ-5D: Presence of problems*															
Mobility	N (%)	56	(52.4)	57	(53.3)	26	(72.2)	30	(42.9)						
Self-care	N (%)	41	(38.3)	29	(27.1)	17	(47.2)	12	(17.1)						
Usual activities	N (%)	85	(79.5)	59	(55.1)	28	(77.8)	30	(42.8)						
Pain/Discomfort	N (%)	81	(75.7)	67	(62.6)	27	(75.0)	39	(55.7)						
Anxiety/depression	N (%)	102	(95.3)	67	(62.7)	35	(97.2)	31	(44.3)						
*EQ-5D-Index*	Mean (SD)	0.7	(0.1)	0.8	(0.1)	0.7	(0.1)	0.8	(0.1)	0.1	(0.1)	−0.002	(0.1)	0.1	(0.1)
	Range score	0.5		0.5		0.3		0.4		0.7		0.5		0.7	
*EQ-VAS*	Mean (SD)	42.0	(18.6)	56.9	(20.6)	43.4	(18.5)	64.3	(17.6)	16.2	(24.2)	5.7	(24.1)	21.7	(22.5)
	Range mm	90		100		90		89		120.00		90.00		120.00	

*EQ-5D* EuroQol Group 5D descriptive system (5-dimension self-report questionnaire)

*EQ-5D Index* Summary index score from EuroQol 5D descriptive

*EQ-VAS* EuroQol Group visual analog scale score

**Table 3:** Associations between change in theEQ-5D Index and EQ-VAS and socio-demographic and health conditions

EQ-5D Index					EQ-VAS			
	Unadjusted		Adjusted model		Unadjusted		Adjusted model	
	β	(95 % CI)	β	95 % CI)	β	95 % CI)	β	(95 % CI)
Depressed at follow-up	−0.118	(−0.169; −0.068)**	−0.116	(−0.167; −0.064)**	−17.33	(−27.04; −7.62)**	−18.34	(−27.85; −8.82)**
*Socio-demographic condition*								
Females	0.018	(−0.041; 0.077)	0.013	(−0.040; 0.066)	−3.44	(−14.39; 7.50)	−4.70	(−14.55; 5.15)
Age pr year	0.001	(−0.003; 0.006)	0.0004	(−0.004; 0.004)	−0.71	(−1.46; 0.04)	−0.67	(−1.41; 0.07)
Education ≥ 10 years	−0.014	(−0.068; 0.039)	−0.027	(−0.077; 0.022)	1.83	(−8.07; 11.74)	−2.78	(−12.08; 6.51)
*Health condition*								
GMHR: poor health at T1	−0.076	(−0.127; −0.025)*	−0.066	(−0.115; −0.017) **	−10.76	(−20.35; −1.18)*	−9.34	(−18.36; −0.32)*
MMSE-NR score at T1	0.004	(−0.004; 0.011)	0.002	(−0.005; 0.010)	1.29	(−0.14; 2.72)	0.60	(−0.82; 2.02)
I-ADL score at T1	0.0004	(−0.004; 0.005)	0.002	(−0.002; 0.006)	−0.34	(−1.13; 0.46)	−0.15	(−0.91; 0.62)
MADRS score at T1	0.0002	(−0.003; 0.003)	0.001	(−0.001; 0.004)	0.43	(−0.11; 0.96)	0.62	(0.11; 1.14)*
Adjusted R^2^ (%)				27.0				27.1

*EQ-5D Index* Summary index score from EuroQol 5D descriptive

*EQ-VAS* EuroQol Group visual analog scale score

*GMHR* General medical health rating scale

*MMSE-NR* Mini-mental state examination-norwegian revised version

*I-ADL* Instrumental activities of daily living

*MADRS* Montgomery-Aasberg depression rating scale

*p-value < 0.05

**p-value < 0.01

### Older adults with remission from depression compared to a reference group without depression

The reference group was significantly younger, and had better education and health (*p* < 0.01); see Table 4. They had significantly less frequently presence of problems with mobility (11/106-10.4 %) (*p* < 0.01), usual activities (22/106-20.7 %) (*p* < 0.01) and anxiety/depression (26/106- 24.5 %) (*p* < 0.05), but not less frequently presence of problems with self-care (10/106 9.4 %) and pain/discomfort (42/106- 39.7 %). The mean (SD) EQ-5D Index and EQ-VAS was higher in the reference group: 0.88 (0.15) and 76.65 (18.00) (*p* < 0.01), respectively. In adjusted linear regression analysis controlling for socio-demographics and health conditions the EQ-5 Index and EQ-VAS were no longer different between the older patients with remission from depression and the reference group of community-living older adults without depression; see Table 5. Poorer HQoL by EQ-5D Index and EQ-VAS was associated with higher age, poor physical health, and higher MADRS score. In addition, a poorer EQ-5D Index was explained by poorer instrumental functioning. Explained adjusted variance in EQ-5D Index and EQ-VAS was 45.1 and 46.2 %, respectively.

**Table 4:** Characteristics of patients with remission of depression at follow-up and reference group and comparison of their characteristics

		Non-depressed		Reference sample		Comparison *p*-value^a^
Number		71	(100)	106		
*Socio-demographic variables*						
Females	N (%)	50	(70.4)	70	(66.0)	n.s.
Age (years)	Mean (SD)	75.4	(6.3)	73.0	(7.9)	**
Education ≥ 10 years	N (%)	31	(46.3)	81	(79.4)	**
*Health condition*						
History of cardiovascular disease	N (%)	48	(67.6)	25	(24.8)	**
GMHR: poor health	N (%)	35	(49.3)	16	(15.1)	**
MMSE-NR score	Mean (SD)	26.9	(3.0)	29.0	(1.1)	**
I-ADL score	Mean (SD)	11.3	(4.9)	9.6	(4.2)	**
History of psychiatric problems	N (%)	53	(74.6)	35	(33.0)	**
MADRS score	Mean (SD)	5.4	(4.5)	2.2	(3.9)	**

^a^χ2-test used for comparison of categorical data and Mann–Whitney test for continuous data

*GMHR* General medical health rating scale

*MMSE-NR* Mini-mental state examination-norwegian revised version

*I-ADL* Instrumental activities of daily living

*MADRS* Montgomery-Aasberg depression rating scale

**p-value < 0.01

**Table 5:** The associations between the degree of QOL (EQ-5D Index and EQ-VAS) and group of older adults (patient with remission vs reference), unadjusted and adjusted for socio-economic and health conditions

EQ-5D Index					EQ-VAS			
	Unadjusted		Adjusted		Unadjusted		Adjusted	
	95 % CI		β	95 % CI	β	95 % CI	β	95 % CI
Patients with remission-	−0.071	(−0.116; −0.026) **	0.006	(−0.037; 0.049)	−11.60	(−17.35; −5.89)**	1.68	(−3.81; 7.17)
*Socio-demographic condition*								
Female	−0.019	(−0.068;0.030)	0.028	(−0.010; 0.066)	−2.21	(−8.52; 4.11)	0.72	(−4.09; 5.54)
Age in years	−0.009	(−0.011;-0.006)**	−0.005	(−0.008; −0.003)**	−0.81	(−1.20; −0.43)**	−0.41	(−0.73; −0.08)**
Education ≥ 10 years	0.060	(0.012; 0.107)*	−0.012	(−0.053; 0.028)	8.04	(1.94; 14.14)**	−1.36	(−6.60; 3.88)
*Health condition*								
GMHR as poor	−0.191	(−0.233;-0.150)**	−0.126	(−0.176; −0.076)**	−26.13	(−31.31; −20.96)**	−19.04	(−25.49;-12.59)**
MMSE score	0.018	(0.009;0.026)**	−0.003	(−0.011; 0.005)	2.79	(1.78;3.79)**	0.53	(−0.50; 1.56)
I-ADL score	−0.016	(−0.021;-0.012)**	−0.005	(−0.010; −0.0002)*	−1.63	(−2.23; −1.04)**	0.07	(−0.56; 0.70)
MADRS score	−0.017	(−0.022;-0.011)**	−0.008	(−0.013; −0.003)**	−2.55	(−3.16;-1.95)**	−1.38	(−2.02;-0.73)**
Adj R^2^ %				45.1				46.2

*EQ-5D Index* Summary index score from EuroQol 5D descriptive

*EQ-VAS* EuroQol group visual analog scale score

*GMHR* General medical health rating scale

*MMSE-NR* Mini-mental state examination-norwegian revised version

*I-ADL* Instrumental activities of daily living

*MADRS* Montgomery-Aasberg depression rating scale

*p-value < 0.05

**p-value < 0.01

## Discussion

To our knowledge, this is the first 1 year follow-up study of health-related quality of life (HQoL) among hospitalized psychogeriatric depressed patients. We found that HQoL by EQ-5D Index and EQ-VAS was more improved in those with remission from depression at follow-up than those without remission, even after adjustment for socio-demographics and baseline health conditions. The HQoL in those with remission at follow-up was comparable with the HQoL in a reference group of older adults when adjusting for differences in socio-demographic and health conditions.

The finding in the present study of a significantly improved EQ-5D Index and EQ-VAS in those with remission compared to those with depression at follow-up is analogous to a previous study of psychogeriatric depressed older out-patients [19] and of depressed adults receiving primary care treatment [35]. However, the previous studies had either no or a shorter follow-up.

In the present study the adjusted explained variance for the regression models was about 27 %, similar to the explained variance in a previous study of EQ-5D Index in adults receiving primary health care treatment [35]. In that study, remission of depression, physical health, depression severity, and ability to work were related to overall EQ-5D Index at a 6 month follow-up [35]. In the recent study of hospitalized depressed patients, as in the previously mentioned study of depressed adults receiving primary health care treatment, the improvement of HQoL was not associated with socio-demographic variables such as gender, age, and education [35].

In adjusted analyses of improved HQoL, we found that improved EQ-VAS score was associated with worse baseline MADRS score, while improved EQ-5D Index at follow-up was not explained by baseline MADRS. We do not have a firm explanation for this difference, but the two HQoL measures are slightly different since the EQ-5D Index uses an algorithm that includes the valuations of the general population sample’s response to the questions asked, while EQ-VAS does not [17]. Older adults with more severe baseline depressive symptoms may experience and report changes of EQ-VAS differently than those with less severe baseline depressive symptoms. In line with this, an observational study of HQoL found that older people with depression at baseline were more likely to report improved HQoL after 12 months compared to those without baseline depression [51]. At least, it is not expected that older adults with more severe depressive baseline symptoms accept their health difficulties, lower their expectations, and adjust their internal standards to level out the discrepancy between the possible and the actual situation to a greater degree (“response-shift”) [52] than those with less severe depressive symptoms [9, 53].

HQoL relates to the perceived effects of both mental and physical health [10, 11], and the present study found that those with good baseline physical health were more likely to have improved EQ-5D Index and EQ-VAS at follow-up compared to those with poor physical health, independent of baseline MADRS score and remission of depression at follow-up. Our result is in line with a longitudinal study of EQ-5D Index in adults receiving primary care treatment [35] and a two-year observational study of community-living older adults reporting that higher physical comorbidity was associated with poorer HQoL at follow-up both in depressed and non-depressed older adults [23].

A simple, direct comparison of EQ-5D Index and EQ-VAS between those with remission and a reference sample of older adults showed that HQoL in the reference sample was better than in the patients with remission. However, those in the reference sample were younger and had higher education, better cognitive and instrumental functioning, better physical health, and lower depression symptom scores on MADRS. Adjusting for these conditions, there was no longer a significant differences in health related QOL by EQ-5D Index or EQ-VAS between the two groups. However, EQ-5DIndex may have a ceiling effect in persons with very good health [54]. The participants in the reference group had better physical health than the patients with remission from depression, but the reference group cannot be characterized as very healthy. Thus, we hold it less likely that a ceiling effect is the cause of our finding of no HQoL difference between the reference group and the patients with remission.

Even if the study has a number of strengths, such as using well-known, internationally accepted diagnostic procedures for depression and having information about variables of potential importance to the outcome, like socio-demographic and medical health variables, some limitations need to be addressed. Firstly, the loss of patients at follow-up (25 %) was large, and could have biased the results. However, those who died or were not followed-up had similar distribution of gender, level of education, history of vascular disease, poor physical health, history of psychiatric disease, and baseline MMSE, IADL, and MADRS scores. Secondly, we do not have detailed information about the treatment given (ie the antidepressant medications, ECT and/or milieu therapy). Thirdly, the reference group was not matched to the group of older adults with remission. In addition to better physical and cognitive health, the reference group was younger and had better education than the patients with remission from depression. The comparison took these differences into account. However, there might be rest confounding which the statistical analyses were not able to handle. In addition, other conditions of importance for HQoL than those we have studied might be of importance; eg major life events, social support, and social coherence [55–57]. Thus, interpretation of the results should be done with caution. Fourthly, due to the recruitment process of the non-depressed participants using advertisements in the local newspaper, home nursing offices, senior centers, and various volunteer organizations, the non-depressed group of participants should not be regarded as representative of the general community-living older people without depression.

Finally, the present study employed the tariffs produced by Dolan et al. [17, 28] to calculate the EQ-5D Index. A Swedish study of depressed adults [35] and Norwegian studies of HQoL in older adults [30, 58] used the same tariffs. It has been questioned if EQ-5D Index calculated by use of valuation of health conditions in general populations is the most appropriate approach [35]. A suggested alternative would have been to base the evaluation of individuals who actually have the health state under study [35]. However, EQ-5D is meant to be a generic HQoL measure and such an approach would make a comparison between groups with different health conditions more difficult. Conversely, the simplicity of the Index may more poorly pick up some aspects of depression that have importance for HQoL compared to a longer, generic HQoL measure that assesses the physical, mental, and social dimensions of HQoL separately. However, the EuroQol Group Questionnaire was used since it is not demanding for older adults with restricted capacity and depression; it is cognitively relatively simple to fill in [54]. Thus, an additional longer HQoL measure was not used, but we analyzed both the data from the EQ-5D Index and the EQ-VAS, giving mainly the same results.

## Conclusion

Older hospital patients with depression who experienced remission one year after admission gained HQoL and their HQoL was comparable with the HQoL in a reference group of older adults without depression when adjusting for differences in socio-demographics and health conditions. These findings are important patient health-outcomes and can have importance for health care planners.

## Abbreviations

EQ-5D Index, summary index of the EuroQol Group EQ-5D descriptive system; EQ-VAS, EuroQol Group visual analog scale score; GMHR, general medical health rating; HRQoL, health-related quality of life; MADRS, Montgomery-Aasberg depression rating scale; MMSE-NR, Mini-Mental-State Exam-Norwegian Revised Version
